# When patients fail UNAIDS’ last 90 - the “failure cascade” beyond 90-90-90 in rural Lesotho, Southern Africa: a *prospective cohort study*

**DOI:** 10.7448/IAS.20.1.21803

**Published:** 2017-07-19

**Authors:** Niklaus Daniel Labhardt, Isaac Ringera, Thabo Ishmael Lejone, Molisana Cheleboi, Sarah Wagner, Josephine Muhairwe, Thomas Klimkait

**Affiliations:** ^a^ Clinical Research Unit, Department Medicine, Swiss Tropical and Public Health Institute, Basel, Switzerland; ^b^ University of Basel, Basel, Switzerland; ^c^ SolidarMed, Country-Programme Lesotho, Swiss Organization for Health in Africa, Maseru, Lesotho; ^d^ Laboratory Services, Butha-Buthe District Hospital, Butha-Buthe, Lesotho; ^e^ Molecular Virology, Department of Biomedicine - Petersplatz, University of Basel, Basel, Switzerland

**Keywords:** 90-90-90, treatment failure, HIV, antiretroviral therapy, cascade, second line, viral load, resistance

## Abstract

**Introduction**: HIV-infected individuals on first-line antiretroviral therapy (ART) in resource-limited settings who do not achieve the last “90” (viral suppression) enter a complex care cascade: enhanced adherence counselling (EAC), repetition of viral load (VL) and switch to second-line ART aiming to achieve resuppression. This study describes the “failure cascade” in patients in Lesotho.

**Methods**: Patients aged ≥16 years on first-line ART at 10 facilities in rural Lesotho received a first-time VL in June 2014. Those with VL ≥80 copies/mL were included in a cohort. The care cascade was assessed at four points: attendance of EAC, result of follow-up VL after EAC, switch to second-line in case of sustained unsuppressed VL and outcome 18 months after the initial unsuppressed VL. Multivariate logistic regression was used to assess predictors of being retained in care with viral resuppression at follow-up.

**Results**: Out of 1563 patients who underwent first-time VL, 138 (8.8%) had unsuppressed VL in June 2014. Out of these, 124 (90%) attended EAC and 116 (84%) had follow-up VL (4 died, 2 transferred out, 11 lost, 5 switched to second-line before follow-up VL). Among the 116 with follow-up VL, 36 (31%) achieved resuppression. Out of the 80 with sustained unsuppressed VL, 58 were switched to second-line, the remaining continued first line. At 18 months’ follow-up in December 2015, out of the initially 138 with unsuppressed VL, 56 (41%) were in care and virally suppressed, 37 (27%) were in care with unsuppressed VL and the remaining 45 (33%) were lost, dead, transferred to another clinic or without documented VL. Achieving viral resuppression after EAC (adjusted odds ratio (aOR): 5.02; 95% confidence interval: 1.14–22.09; *p* = 0.033) and being switched to second-line in case of sustained viremia after EAC (aOR: 7.17; 1.90–27.04; *p* = 0.004) were associated with being retained in care and virally suppressed at 18 months of follow-up. Age, gender, education, time on ART and level of VL were not associated.

**Conclusions**: In this study in rural Lesotho, outcomes along the “failure cascade” were poor. To improve outcomes in this vulnerable patient group who fails the last “90”, programmes need to focus on timely EAC and switch to second line for cases with continuous viremia despite EAC.

## Introduction

Achievements over the last few years ignited hope; the HIV burden in sub-Saharan Africa could fall to numbers small enough to trigger a turn in the epidemic and finally towards solving the global HIV health crisis [1]. Provision of antiretroviral therapy (ART) to all persons infected with HIV is a key element of the UNAIDS strategy 2016–2021 [2]. Along with the roll-out of ART, the term “HIV care cascade” gained importance. It is used to report on the effectiveness of HIV programmes, commonly defined as the steps HIV-infected individuals must take along a “continuum of HIV care” towards achieving viral suppression through ART [3]. Eventually, the 90-90-90 targets published by the Joint United Nations Programme on HIV/AIDS (UNAIDS) followed the logic of this care continuum focusing on the three crucial steps in the cascade: diagnosis of HIV infection, linkage to HIV care with sustained provision of ART and viral suppression through ART [4]. However, individuals may get lost at every step of this care cascade, which can substantially diminish the overall effectiveness of HIV programmes [5,6]. Accordingly, major efforts are currently invested in improving coverage of HIV testing, subsequent linkage to care, initiation of and retention on ART [7].

**Figure 1.**
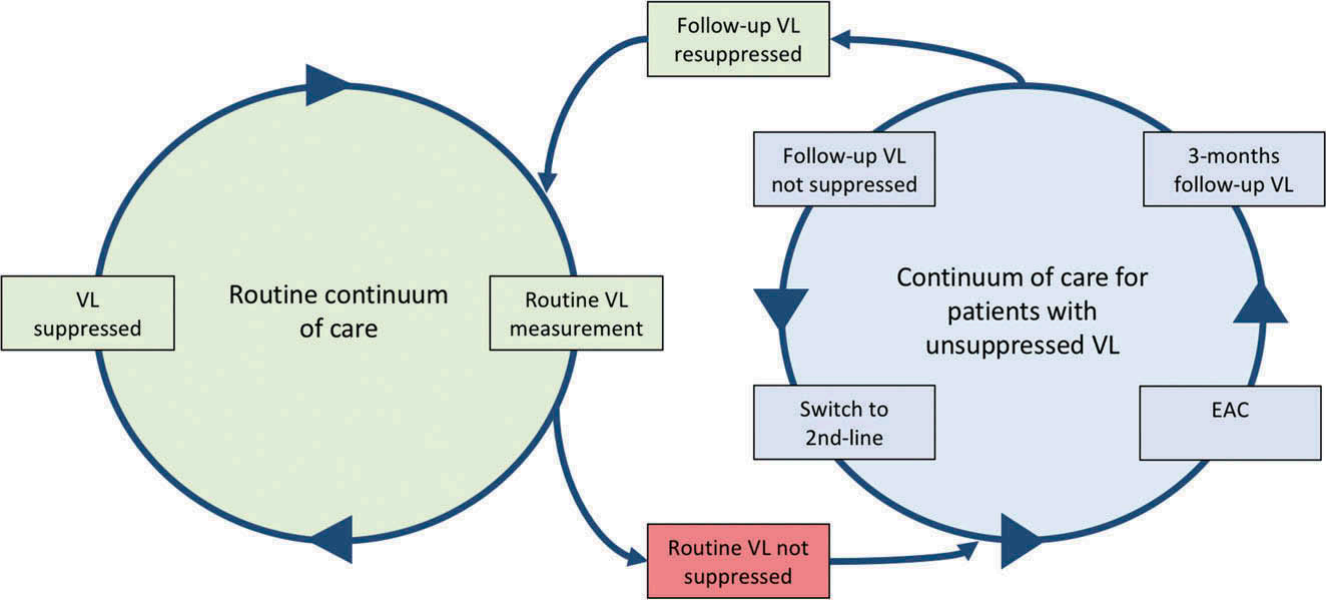
The routine continuum of care for individuals on antiretroviral therapy (ART) and continuum of care for patients with unsuppressed VL. VL: viral load; EAC: enhanced adherence counselling.

However, those patients, who managed the first two steps of the cascade (HIV testing and linkage to care) but subsequently do not achieve the third step, viral suppression, enter a new cascade that currently appears to be rarely assessed or reported. The events of such a “failure-cascade” for individuals with unsuppressed viral load (VL) are challenging to patients and often understaffed and poorly resourced HIV care facilities in sub-Saharan Africa. Depending on setting and thresholds, about 15% of all patients taking first-line ART in resource-limited settings do not achieve viral suppression [8]. The World Health Organization (WHO) thus recommends concrete management steps in case of unsuppressed VL [9]: First, the patients are informed about the test result and receive enhanced adherence counselling (EAC); three to six months after the first VL, they receive a second VL measurement. If at this follow-up the VL is resuppressed, the patient will continue first-line ART and stay in the routine monitoring schedule with 6- or 12-monthly VL testing, depending on local guidelines and resources. Patients with an unsuppressed follow-up VL despite improved adherence qualify for a switch to second-line ART. Once on second-line ART, viral resuppression must be achieved before the patient can enter again the routine monitoring cycle. The different steps for patients with unsuppressed VL are detailed in Figure 1. Patients may get lost from care at each step.

To our knowledge, there are currently no reports from rural sub-Saharan Africa that describe the full cascade from a first unsuppressed VL to resuppression under the second-line regimen. However, several studies reporting on parts of this cascade imply that the continuum of care for patients with unsuppressed VL is particularly vulnerable. In a study in Swaziland, Jobanputra and colleagues report that only 60% of patients with a first unsuppressed VL had a documented follow-up VL within 6 months [10]. And even where patients had received a follow-up VL, only a minority of those with continuous virologic failure was switched to a second-line regimen in a large urban South African treatment programme - although guidelines strongly recommended the switch for precisely these cases with two consecutive elevated VLs [11]. Furthermore, among those who were switched, the proportion of patients achieving full viral resuppression with the new regimen remained often low. Studies report treatment success rates varying between 48% and 72% [12–14].

In this paper, we describe the full failure cascade from a first unsuppressed VL to resuppression after switch to second-line ART in a registered prospective multicentre cohort study in 10 rural clinics in Lesotho, Southern Africa.

## Methods

### Study design

The registered prospective study entitled “Comorbidities and Virologic Outcomes Among Patients on Antiretroviral Therapy in Rural Lesotho” (CART-1 study) assessed virologic outcomes among HIV-infected patients on first-line ART in 10 rural facilities in Lesotho (www.clinicaltrials.gov; ID: NCT02126696). Assessment of the WHO-recommended follow-up algorithm for patients with unsuppressed VL was one of the two registered primary objectives of the CART-1 study. In May/June 2014, a total of 1563 adult patients on non-nucleosidic reverse-transcriptase inhibitor (NNRTI)-based first-line ART without any previous access to VL monitoring received a first VL and in the case of unsuppressed VL EAC and a follow-up VL after 3 months (October 2014). As per guidelines, those patients with continuously unsuppressed VL qualified for switch to second-line ART [15]. Outcomes of first and follow-up VL including resistance testing have previously been reported by our group [16].

**Figure 2.**
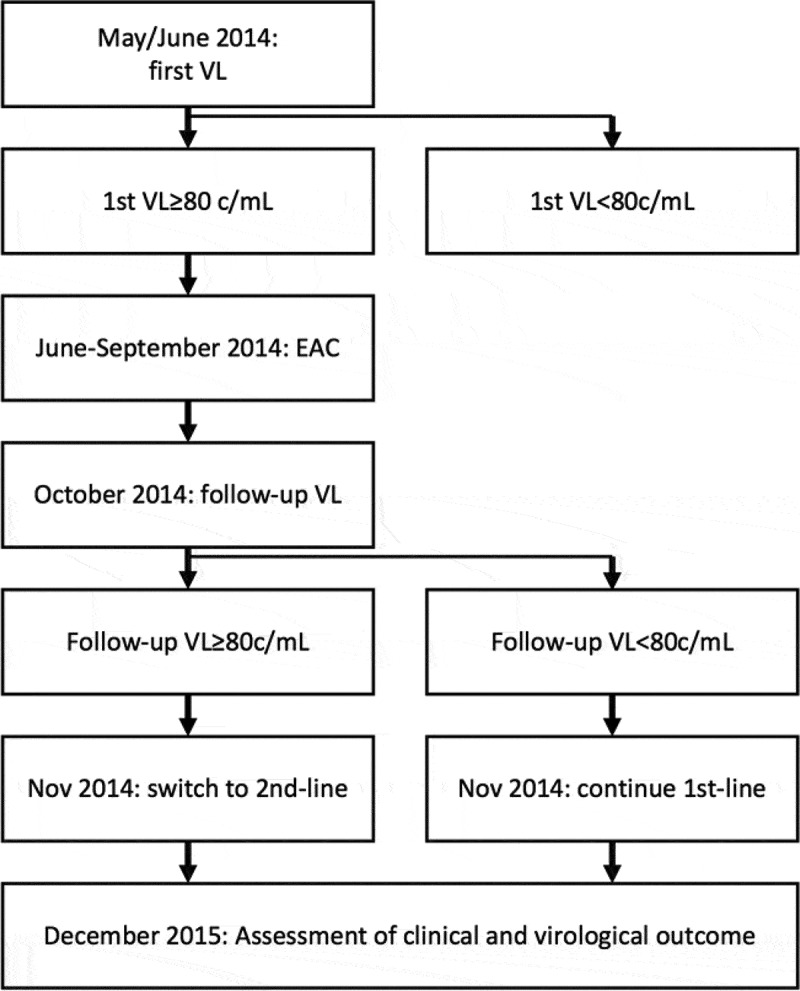
Study flow and time-points of assessment. VL: viral load; EAC: enhanced adherence counselling; c/mL: copies/mL.

The main objective of this work was to analyse the 18 months’ follow-up data of the 138 patients with unsuppressed VL in May/June 2014 and to describe their respective care cascade as outlined in Figure 2. As secondary objectives, we provide subgroup analyses, assessment of predictors of a favourable outcome at 18 months of follow-up and genotypic resistance data on individuals who were unable to suppress VL under second-line ART by December 2015.

### Study setting

Participants were recruited in two hospitals and eight health centres in two districts in Lesotho, Thaba-Tseka and Butha-Buthe. Lesotho, a small landlocked country surrounded by South Africa, has an adult HIV prevalence of 25% [17]. In the 10 study clinics, HIV care is exclusively being provided by trained nurses. All sites receive support through SolidarMed, a Swiss not-for-profit organization that has been assisting the Ministry of Health in the roll-out of ART in Lesotho since 2005. Details of the setting have been described previously [18]. Even though Lesotho National Guidelines recommend VL monitoring since 2013 [19], routine VL monitoring was not available in 2014 when the study started. Before enrolment in the study, participating patients had no access to VL testing and were monitored exclusively based on CD4 cell count and clinical assessment.

### Participants and study procedure

Eligibility criterion was prior continuous NNRTI-based first-line ART for ≥6 months. Exclusion criteria were any shorter period on ART, documented treatment interruption of ≥7 consecutive days during the last 3 months or being on a protease inhibitor-based regimen. Patients aged ≥16 years were included in this analysis. Based on the specifications of our validated test system, patients with VL above 80 copies/mL were defined as “unsuppressed”. Outcomes of first VL measurement have been reported elsewhere [20].

Figure 2 displays the study flow: Participants were recruited between May and June 2014 for a first VL measurement. In June/July 2014, every patient with VL ≥80 copies/mL was informed about the unsuppressed VL via their healthcare facility and invited for EAC. As per guidelines, a follow-up VL was taken 3 months thereafter (October 2014). In November 2014, facilities were informed about the result of this follow-up VL, and in case of sustained unsuppressed VL, a second-line regimen, based on the results from genotypic resistance testing and the availability of drugs, was recommended. In December 2015, study nurses assessed the outcomes of all 138 patients with a first unsuppressed VL in May/June 2014. In case of unsuppressed VL, genotypic sequencing was performed.

### Data collection and processing

Three time points served for data collection: (1) May/June 2014 baseline data and the first VL sample were collected, (2) October 2014 information on attendance of EAC and follow-up VL sample were collected and (3) in December 2015, 18 months’ clinical outcome and VL sample were collected. Patients’ baseline characteristics were recorded at the time when blood was drawn for the first VL measurement in May/June 2014. Using a structured questionnaire, trained, supervised lay counsellors interviewed participants on social and demographic characteristics, and a trained ART nurse recorded clinical and therapeutic information. Questionnaires were digitalized at the Butha-Buthe hospital data centre in Lesotho and subsequently processed with Data-Scan 5.7.7 (Neoptec, Montpellier, France) for electronic data capture. Prior to analysis, all data were manually cross-checked against the original records. For follow-up data of patients with unsuppressed VL (October 2014 and December 2015), study nurses recorded clinical information on paper-based case-reporting forms. Data were subsequently entered into a database, using double-data entry to ensure accuracy.

Routine laboratory exams (full blood count, CD4 cell count, transaminases and serum creatinine) were performed at the nationally certified laboratories of Butha-Buthe Hospital and Thaba-Tseka Hospital. For first and follow-up VL in 2014, venous blood was collected in Cell Preparation tubes (CPT) and centrifuged and frozen at −80 degrees within six hours. The samples were subsequently sent on dry ice to a reference laboratory in Switzerland. The VL analyses for December 2015 were performed on a newly installed and accredited platform in Butha-Buthe Laboratory (COBAS® AmpliPrep/COBAS® TaqMan® HIV-1 Test, v2.0, Roche Diagnostics, Johannesburg, South Africa). In addition to these, for quality assurance, 20% of samples were transported on dry ice to a reference laboratory in Switzerland, similar to the analysis in 2014. Using the cut-off at 80 copies/mL, a 100% agreement for suppressed versus unsuppressed VL was found between the two laboratories. Genotypic resistance testing was conducted for follow-up samples with unsuppressed VL (≥80 copies/mL) after EAC in October 2014, using NucliSENS easyMag extraction of viral RNA from plasma. In case sequencing was not successful, that is, in case of low-level viremia, the sample from first VL determination (May/June 2014) was used. This was the case in two patients. Results from sequencing were classified according to the HIV Drug Resistance Database of Stanford University (http://hivdb.stanford.edu).

### Outcomes measured and statistical analysis

The primary objective of this analysis was to describe the failure cascade for patients with a first-time determination of an unsuppressed VL. Description of the cascade followed the logic of the “continuum of care” for patients with unsuppressed VL as displayed in Figures 1 and 2. The clinical outcome in December 2015 was categorized in “retained in care with viral suppression” (VL <80 copies/mL), “retained in care without viral suppression”, “retained in care without documentation of VL”, “dead”, “transferred out” or “lost to follow-up” (LTFU). To ascertain the clinical outcome, all patients retained in care were invited for follow-up VL testing, and study nurses traced patients who did not attend for this follow-up VL. Tracing was done via phone - if available - or via a village health worker who visited the patient’s home. Patients not found through tracing were categorized as “dead” if a family member or the village-chief confirmed the death, “transferred out” if a written confirmation of the patient being in care at another facility was available or “LTFU” if the patient’s status of care could not be ascertained.

In Table 3, a second-line regimen was labelled as “partially active” if genotypic resistance testing revealed at least “low-level resistance” according to the HIV Drug Resistance Database of Stanford University against at least two drugs of the second-line regimen. On the other hand, the second-line regimen was labelled “fully active” if genotypic resistance testing did not reveal any major resistance mutations against at least two drugs of the second-line regimen. To assess potential predictors of viral suppression 18 months after the first unsuppressed VL (Table 4), the patients’ outcome was categorized into “retained and virally suppressed” and “not retained or not suppressed”, which included all other potential outcomes (dead, LTFU, transferred out, unsuppressed VL or no documented VL). Patient characteristics were analysed for a potential association with “being retained and virally suppressed” using univariate logistic regression. Variables with a potential association at significance level <0.2 were subsequently fed into a multivariate logistic regression model reporting adjusted odds ratios (ORs). Household wealth quintiles of patients were derived from a wealth index that was generated through principal component analysis [21]. For the co-variate “resistance against first line” in Table 4, a drug was considered “active” if, according to the HIV Drug Resistance Database of Stanford University, genotyping revealed “susceptible” or “potential low-level resistance”.

**Table 1.** Baseline characteristics of the 138 patients with unsuppressed VL in June 2014

Clinical characteristics	
Median age (IQR)	41.1 (32.4–49.9)
Female gender (%)	91 (65.9)
Median time on ART (years) (IQR)	4.1 (2.4–5.7)
First-line regimen’s NRTI backbone	
● Zidovudine/lamivudine (%)	70 (50.7)
● Tenofovir/lamivudine (%)	67 (48.6)
● Abacavir/lamivudine (%)	1 (0.7)
First-line regimen’s NNRTI	
● Efavirenz (%)	90 (65.2)
● Nevirapine (%)	48 (34.8)
Median viral load May/June 2014 (copies/mL; IQR)	7496 (1447–21,824)
Median CD4 cell count May/June 2014 (cells/µL; IQR)	351 (182–520)

IQR: interquartile range; NRTI: nucleoside reverse-transcriptase inhibitor; NNRTI: non-nucleoside reverse-transcriptase inhibitor; VL: viral load; ART: antiretroviral therapy.

**Table 2.** Stratified outcomes of 138 adult patients on first-line ART 18 months after a first measurement of an unsuppressed VL

Subgroup	*N*	VL <80 c/mL	VL ≥80 c/mL	Died	LTFU	In care, no VL result
First and follow-up VL ≥80 c/mL	80	34 (42.5)	24 (30.0)	3 (3.8)	14 (17.5)	5 (6.3)
● Switched to second line	58	32 (55.2)	13 (22.4)	2 (3.6)	8(13.8)	3 (5.2)
● Continued first line	22	2 (9.1)	11 (50.0)	1 (4.6)	6 (27.3)	2 (9.1)
First VL ≥80 c/mL, follow-up VL <80 c/mL continued first line	36	20 (55.6)	7 (19.4)	1 (2.8)	7 (19.4)	1 (2.8)
First VL ≥80 and switched to second line	5	0 (0.0)	2 (40.0)	2 (40.0)	1 (20.0)	0 (0.0)
First VL ≥80 c/mL, no follow-up VL, continued first line	17	2 (11.8)	4 (23.5)	4 (23.5)	7 (41.2)	0 (0.0)
All patients with first VL ≥80 c/mL	138	56 (57.9)	37 (26.8)	10 (7.2)	29 (21.0)	6 (4.3)

VL: viral load; c/mL: copies/mL; LTFU: lost to follow-up; ART: antiretroviral therapy.

**Table 3.** Outcomes of the 58 patients switched to second line after second unsuppressed VL, stratified by activity of their second-line regimen as determined by pre-switch genotyping

	*N*	VL <80 copies/mL	VL ≥80 c/mL	Died	LTFU	In care, no VL result
Switched to second line	**58**					
● Fully active second-line regimen	35	14 (40.0)	11 (31.4)	2 (5.7)	6 (17.1)	2 (5.7)
● Partially active second-line regimen	23	18 (78.3)	2 (8.7)	0 (0.0)	2 (8.7)	1 (4.4)

VL: viral load; c/mL: copies/mL; LTFU: lost to follow-up.

**Table 4.** Association between patient characteristics and being retained in care with viral resuppression at 18 months’ follow-up

	*N* (%)	Viral resuppression, *N* (%)	OR (95% CI)	*p*-Value	aOR (95% CI)	Adjusted *p*-value
**Gender**					-	-
● Male	47 (34)	19 (40)	1			
● Female	91 (66)	37 (41)	1.01 (0.49–2.07)	0.979		
**Age**					-	-
● 16–34 years	45 (33)	17 (38)	1			
● ≥35 years	93 (67)	39 (42)	1.19 (0.57–2.47)	0.641		
**Travel time to facility**^a^			0.94 (0.69–1.28)	0.700	-	-
● 0–0.5 h	27 (20)	11 (41)				
● 0.5–1 h	37 (27)	15 (41)				
● 1–2 h	32 (24)	17 (53)				
● ≥2 h	39 (29)	13 (33)				
**Household wealth quintile^b^**			0.96 (0.75–1.22)	0.714	-	-
**Education^c^**					-	-
● No primary education	74 (54)	29 (39)	1			
● Primary education and higher	63 (46)	27 (43)	1.16 (0.59–2.30)	0.663		
**Time since ART started^d^**			1.19 (0.89–1.62)	0.240		
● <2 years	30 (23)	8 (26)				
● 2–3.5 years	18 (14)	10 (56)				
● 3.5–5 years	36 (27)	14 (39)				
● >5 years	49 (37)	22 (45)				
**VL in May/June 2014**						
● 80–999 copies/mL	28 (20)	17 (61)	1		1	
● ≥1000 copies/mL	110 (80)	39 (35)	0.36 (0.15–0.83)	0.017	0.46 (0.14–1.48)	0.191
**VL October 2014 after EAC^e^**						
● ≥80 copies/mL	80 (69)	34 (43)	1		1	
● <80 copies/mL	36 (31)	20 (56)	1.69 (0.77–3.74)	0.194	5.02 (1.14–22.09)	0.033
**VL October 2014 after EAC^e^**						
● ≥1000 copies/mL	67 (57)	31 (46)	1		-	
● <1000 copies/mL	51 (43)	23 (45)	0.95 (0.46–1.98)	0.899		
**Resistance against first line^f^**						
● ≥2 drugs still active	50 (43)	21 (42)	1			
● <2 drugs active	66 (57)	33 (50)	1.38 (0.66–2.89)	0.393		
**Switched to** second **line**						
● No	75 (54)	24 (32)	1		1	
● Yes	63 (46)	32 (51)	1.98 (0.99–3.94)	0.052	7.17 (1.90–27.04)	0.004
**Facility type**						
● Hospital	61 (44)	29 (48)	1		1	
● Health centre	77 (56)	27 (35)	0.59 (0.29–1.18)	0.140	0.66 (0.29–1.47)	0.309

All 138 patients with unsuppressed VL at first measurement in May/June 2014 are included.

**Figure 3.**
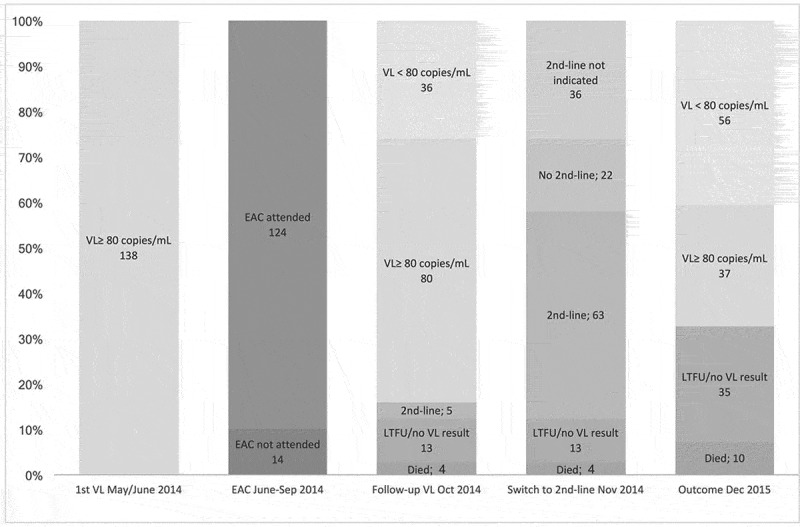
The failure cascade in 138 patients with a first-time measurement of an unsuppressed viral load. VL: viral load; EAC: enhanced adherence counselling; TO: transferred out; LTFU: lost to follow-up.

**Figure 4.**
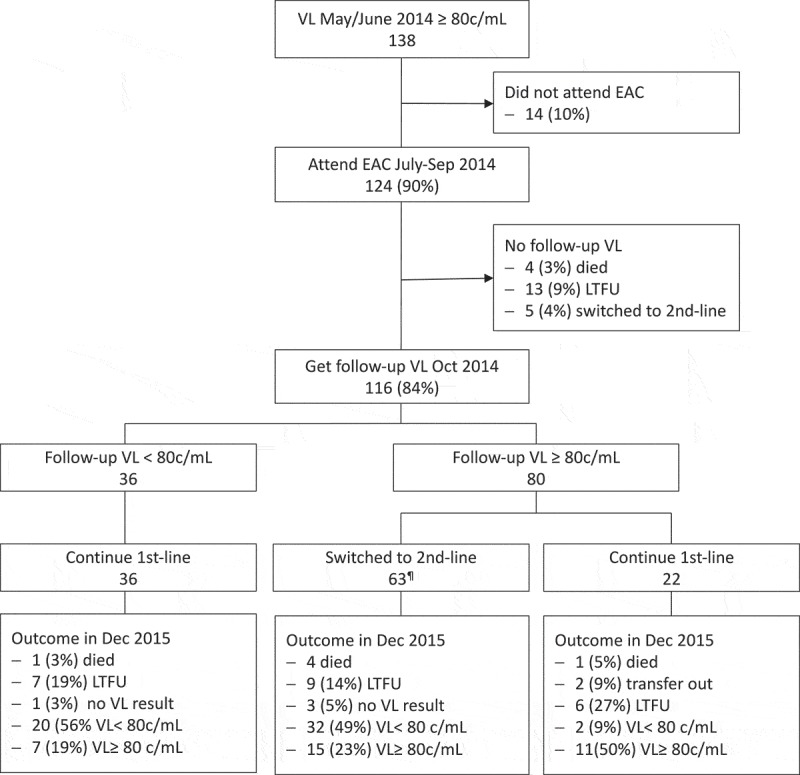
The failure cascade in 138 patients with first-time unsuppressed VL while taking first-line ART. Outcomes are stratified by result of follow-up VL after EAC and if patients were switched to second-line or not. ¶ Includes the 5 patients already switched after first VL in May/June 2014. VL, viral load; EAC, enhanced adherence counseling; c/mL, copies/mL; LTFU, lost to follow-up.

^a^Three missing values. ^b^Per quintile, 1 being lowest and 5 highest household wealth quintile. **^c^**One missing value for the variable education. ^d^Five missing values for time since ART started. ^e^22 had no VL after EAC, see Figures 3 and 4. ^f^Only includes the 116 patients with follow-up VL after EAC (see Figure 3). OR: odds ratio; aOR: adjusted odds ratio; CI: confidence interval; VL: viral load; ART: antiretroviral therapy; EAC: enhanced adherence counselling.

Data on genotypic results from patients who were switched to second line and did not achieve viral suppression by December 2015 (supplement 1) are only presented descriptively due to the small sample size.

### Ethics statement

Ethics approval for the study was received from the National Health Research and Ethics Committee of Lesotho (ID 01-2014) and the “Ethikkomission Nordwest- und Zentralschweiz” (EKNZ) in Switzerland (ID 2014-029). Prior to enrolment, all patients provided individual written informed consent. Patients and their healthcare providers were informed within 2 weeks about VL results and genotypic resistance results in case of unsuppressed VL.

## Results

### Cascade after unsuppressed VL

Table 1 displays characteristics of the 138 patients presenting with unsuppressed VL in June 2014. Figures 3 and 4 show the care cascade of these patients following the first unsuppressed VL: 124 (90%) received at least one adherence intervention (113 (85%) ≥1 adherence focus group discussion, 69 (52%) ≥1 one-to-one adherence counselling and 35 (26%) directly observed therapy through village health worker). Thereafter, 116 (84%) received the 3-month follow-up VL in October 2014. Reasons for not attending were death (3 AIDS-related, 1 non-AIDS related), transfer to another clinic (2), not attending the clinic (7 LTFU, 4 at work in South Africa) or switched to second line after first VL (5). Among those with follow-up VL, 36 (31%) had achieved viral resuppression; the remaining 80 continued to present VLs ≥80 copies/mL. Those with resuppression after EAC had a significantly lower median VL at baseline (433 copies/mL, IQR: 157–11,556) than those with sustained viremia (12,602 copies/mL; IQR: 3948–26,870) (*p* < 0.001).

Out of the 80 patients with sustained viremia after EAC, only 58 (73%) were switched to second-line ART and the remaining continued first-line therapy. Patients with VL ≥1000 copies/mL after EAC were more likely to be switched than patients with VL 80–999 copies/mL (82% versus 29%, OR: 11.3 (95% confidence interval (CI): 3.0–42.0; *p* < 0.001)).

At follow-up in December 2015, out of the originally 138 with unsuppressed VL in June 2014, 56 (41%) were still in care and virally suppressed, 37 (27%) were retained in care with unsuppressed VL and the remaining 45 (33%) were LTFU, confirmed dead, transferred to another clinic or had no documented VL result.

### Subgroup analyses

Table 2 displays outcomes stratified by subgroup. Among those 58 individuals who were managed as per national and international guidelines, that is, directly switched to second line after two consecutive unsuppressed VL, all received ritonavir-boosted lopinavir (LPV/r) plus TDF/3TC (32 (55%)), AZT/3TC (21 (36.2%)), ABC/3TC (1(2%)) or raltegravir (4 (7%)). At follow-up in December 2015, out of the 58 patients switched to second line, 32 (55%) were found to be retained in care and virally suppressed. There were no significant differences of outcomes across the different second-line regimens.

However, as shown in Table 3, patient switched to a second-line regimen with only partial activity were more likely to be retained and virally suppressed compared to patients switched to a fully active second-line regimen (18/23 versus 14/35, OR: 5.4 (95% CI: 1.6–17.9); *p* = 0.006).

Out of the 26 patients who did not achieve viral suppression after switch to second line, 8 had the complete information available for pre- and post-switch genotypic resistance and for relevant clinical parameters. None had developed major protease inhibitor resistance mutations (supplement 1).

Among the 22 who were - against the guidelines - not switched to second line despite two consecutive unsuppressed VL in 2014, only 2 (9%) had achieved viral resuppression by December 2015. Among the 36 who were initially virologically unsuppressed but then resuppressed at the follow-up VL test in 2014 after EAC, 20 (56%) were still retained in care with suppressed VL by December 2015 (Table 2, Figure 4).

### Predictors of viral resuppression

Out of the total of 138 individuals with unsuppressed VL in June 2014, 56 (41%) were retained in care with documented viral suppression by December 2015. The only two variables significantly associated with “being retained in care and virally suppressed” were (1) to have achieved viral resuppression on first-line ART after EAC in October 2014 and (2) being switched to second line in case of a second unsuppressed VL in October 2014 (see Table 4). Socio-demographic variables, time on ART, pre-switch resistance to first-line ART and level of pre-switch VL were not associated to viral suppression in December 2015 (Table 4).

## Discussion

In this registered prospective cohort study, we describe the care cascade of adult HIV-infected individuals on first-line ART who have unsuppressed VL in 10 clinics in rural Lesotho. Contrarily to other studies, we looked at the whole “failure cascade” from first unsuppressed VL to EAC, follow-up VL, switch to second line in case of sustained unsuppressed viremia and virologic outcome after switch. Considering this whole cascade, 18 months after first-time determination of unsuppressed VL, only 41% were found to still be retained in care with documented viral resuppression (Figures 3 and 4). This poor outcome underlines the high vulnerability of the specific patient subgroup of this study and may reflect the real-life care cascade for patients with unsuppressed viremia in resource-limited settings. While national programmes focus on the three 90ies of the UNAIDS strategy [2], it will be absolutely crucial to address the particular needs of patients who fail to achieve the third 90, which is viral suppression. We found no association between socio-demographic variables, treatment history, viral resistance and viral resuppression at 18 month follow-up. In multivariate analysis, only two factors were significantly associated to being retained and virally suppressed at 18 month follow-up: achieving resuppression after EAC and being switched to second-line ART in case of sustained unsuppressed VL after EAC. This underlines timely adherence interventions and switch to second line in case of sustained failure as key components to improve outcomes in this patient group. Among the 10 targets set by UNAIDS in its strategy, there is none that would specifically address a better management of patients with unsuccessful therapy - be it for reasons of poor adherence or HIV resistance [2]. However, along with the massive scale-up of ART coverage in high-prevalence settings, the numbers of patients failing to suppress viremia while taking ART are expected to rise and, along with it, a considerable threat by emerging HIV resistances and continuing transmission despite high ART coverage [22].

Patients fail to control their VL again for different reasons: Some of the poor outcomes may be attributed to a fragile and underresourced healthcare system. As shown in Figures 3 and 4, a considerable number of patients were lost to follow-up already after the first VL determination. Good record-keeping and intensive tracking of patients with unsuppressed VL might be suitable tools for improving retention in care [23]. Another reason for continuously detectable VLs, driven by weak health care, is the failure to follow guidelines and to appropriately switch patients to second line promptly when they qualify for this change. Even though our study procedures included oral and written communication to the health facility about every patient qualifying for second-line ART with recommendation of the new regimen of choice, more than a quarter of patients did not benefit from a switch to second-line ART. One reason may be that nurses did not want to switch patients with sustained unsuppressed VL but below 1000 copies/mL. However, also among those with VL≥ 1000 copies, one out of five patients was not switched to second line. The exact reasons are not clear, but one likely driver is that nurses were hesitant to switch a patient to a regimen, with which they were personally not very familiar. In a previous study, we already observed this phenomenon at the time when tenofovir was introduced as the preferred first-line regimen in Lesotho: It took nurse-led facilities longer to adopt these guidelines and to prescribe the newly recommended regimen to patients starting ART [24]. Eventually, the high level of task-shifting and decentralization of ART provision to all nurse-led health centres in Lesotho had been a main reason for the successful scale-up of ART coverage [25]. However, this model may still be very vulnerable when it comes to first-line failures and switching to novel antiretroviral drugs. Now, based on the roll-out of VL monitoring, more patients with current treatment failures will be identified. Accordingly, training and mentoring of ART nurses on diagnosis and management of treatment failure must become a priority for countries with a high degree of task shifting and decentralization. In agreement with our study, Johnston and colleagues had previously shown for a large multicentre cohort in Johannesburg a high reluctance among healthcare providers to switch patients to second-line ART - even after confirmed treatment failure [11]. Rohr and colleagues reported from a large cohort of nine clinics in South Africa that only about half of the patients failing first-line therapy were switched to second line within 12 months after the diagnosis of treatment failure [26]. These South African studies were conducted in large, well-established HIV care centres. The low rate of switch to second line there may indicate that providers’ principal hesitance to change to second line may not only relate to a lack of experience but other factors, such as assumed poor adherence or the general reluctance to “waste” second-line options prematurely. Qualitative studies that specifically assess providers’ reasons for not switching patients who fail first-line therapy will be needed to design targeted intervention programmes.

Yet, the failure to resuppress viral replication was not only due to poorly managed attrition from care or missed switching to second line. Also, among those 58 patients, who were correctly switched after two consecutive unsuppressed VL, we observed that only 32 (55%) of those retained in care were suppressed at month 18 of follow-up (Table 2, Figure 4). These figures are in line with earlier reports from South Africa [12,27]. In the cohort study of Rohr et al., after switch to second line, 14% experienced confirmed virologic failure on second line, 42% remained in care with regular VL monitoring, 12% remained in care but had missed VL monitoring visits, 30% stopped attending the clinic and 2% died [26]. In a randomized controlled trial conducted in three West African countries, 65% achieved viral suppression (<50 copies/mL) after switch to second line [28].

For unexplored reasons, outcomes in Asian ART programmes seem to achieve superior outcomes: In a cohort of 302 patients switched to second line in 12 Asian countries, the rate of treatment failure and mortality per 100 patient/years was 8.8 [29]. However, Chakravarty and colleagues reported 60% to be in care and virally suppressed (<400 copies/mL) at one year after switch to second line under programmatic conditions in India [30].

Similar to other studies, patients in our study, who were switched to an only partly active second-line regimen, had paradoxically better outcomes than patients switched to technically fully active regimens [31–34]; this could indicate a continuing poor adherence as main reason for persistent viremia. As summarized in supplement 1, none of the patients with available pre- and post-switch genotypic resistance information had developed any major resistance mutations to the protease inhibitor of his/her respective regimen. In line with this, most studies from sub-Saharan Africa report no detected protease inhibitor HIV mutations in patients failing second-line ART [27]. This good protease inhibitor performance, however, could be due to the relatively short follow-up periods: Rawizza and colleagues have observed a notable accumulation of protease inhibitor resistance mutations 24 months after switch to second line in a significant proportion of patients in Nigeria [35].

In our study, having detectable VLs at a low (80–999 copies/mL) versus high level (≥1000 copies/mL) after EAC in October 2014 was not associated with better outcomes in December 2015 (Table 4). This underlines that sustained viremia, even if below the WHO-recommended cut-off of 1000 copies, should be considered for switching to second line. In earlier work, we demonstrated that patients with continuous VL levels between 80 and 999 copies were as likely to present therapy-relevant resistance mutations as patients with VL ≥1000 copies/mL [16].

## Limitations

Our study has several limitations. First, the surprisingly low number of patients with virologic failure at the first measurement in May/June 2014 yielded a low overall sample size, which limits the statistical robustness of the assessment of predictors for viral resuppression in this cohort. Second, genotypic resistance testing was available for only a small number of patients who failed to resuppress HIV under second-line ART. Third, although our setting, which includes very remote health centres in the Lesotho mountains, well reflects the reality of ART care in rural Africa, the crucial regular support by a very active non-governmental organization (NGO), SolidarMed, renders these centres privileged compared to regions without any support from NGOs. This implies that outcomes in other settings may even be significantly poorer than shown in our study. Fourth, we have no information on possibly reasons why patients were not switched to second-line ART.

## Conclusions

At the end of the 90-90-90 cascade, patients who fail to achieve viral suppression while taking ART enter the “failure care cascade” on the way to viral resuppression. In our study, conducted in nurse-led facilities in rural Lesotho, overall outcome of this cascade was poor with only 41% being in care with resuppressed VL at 18 months’ follow-up. Substantial numbers of patients were lost at each step along the continuum of care leading from first detection of unsuppressed VL to attendance of EAC, follow-up VL, switch to second-line ART and viral resuppression under the new second-line regimen. Achieving viral resuppression after adherence counselling and being switched to second line in case of ongoing viremia despite adherence counselling were associated with a favourable outcome at 18 months’ follow-up. In parallel to further scaling up of first-line ART provision, HIV programmes must develop strategies and allocate resources for those patients who at the end of the 90-90-90 cascade cannot achieve viral suppression and enter the “failure care cascade”. Ensuring timely EAC and switch to second-line ART in case of sustained unsuppressed VL are key for this vulnerable group of patients.

## Supplemental Material

To access the supplementary material to this article please see Supplementary Files under Article Tools online.

## References

[1] UNAIDS How AIDS changed everything; MDG 6: 15 years, 15 lessons of hope from the AIDS response. 2015 Available from: www.unaids.org

[2] UNAIDS On the fast-track to end AIDS: UNAIDS 2016–2021 strategy. 2016 [cited 2016 104] Available from: http://www.unaids.org/sites/default/files/media_asset/20151027_UNAIDS_PCB37_15_18_EN_rev1.pdf

[3] Hull M, Lange J, Montaner JSG. Treatment as prevention - where next? Curr HIV/AIDS Rep. 2014 12;11(4):496–10. 25384357 10.1007/s11904-014-0237-5 PMC4268430

[4] 90-90-90 an ambitious treatment target to help end the AIDS epidemic. UNAIDS/JC2684. 2014 10 [cited 2014 Oct 30] Available from: www.unaids.org

[5] Govindasamy D, Kranzer K, Ford N Strengthening the HIV cascade to ensure an effective future ART response in sub-Saharan Africa. Trans R Soc Trop Med Hyg. 2014 1;108(1):1–3. 24284954 10.1093/trstmh/trt105

[6] MacCarthy S, Hoffmann M, Ferguson L, Nunn A, Irvin R, Bangsberg D, et al The HIV care cascade: models, measures and moving forward. J Int AIDS Soc. 2015;18(1):19395. 25735869 10.7448/IAS.18.1.19395 PMC4348400

[7] UNAIDS Fast track - ending the AIDS epidemic by 2030. UNAIDS/JC2686. 2014 [cited 2015 718] Available from: http://www.unaids.org/sites/default/files/media_asset/JC2686_WAD2014report_en.pdf

[8] McMahon JH, Elliott JH, Bertagnolio S, Kubiak R, Jordan MR Viral suppression after 12 months of antiretroviral therapy in low- and middle-income countries: a systematic review. Bull World Health Organ. 2013 5 1;91(5):377–385E. 23678201 10.2471/BLT.12.112946 PMC3646348

[9] World Health Organization Consolidated guidelines on the use of antiretroviral drugs for treating and preventing HIV infection - recommendation for a public health approach. 2nd ed. 2016 6 [cited 2016 Aug 15] Available from: http://apps.who.int/iris/bitstream/10665/208825/1/9789241549684_eng.pdf?ua=1 27466667

[10] Jobanputra K, Parker LA, Azih C, Okello V, Maphalala G, Kershberger B, et al Factors associated with virological failure and suppression after enhanced adherence counselling, in children, adolescents and adults on antiretroviral therapy for HIV in Swaziland. PLoS One. 2015;10(2):e0116144. 25695494 10.1371/journal.pone.0116144 PMC4335028

[11] Johnston V, Fielding KL, Charalambous S, Churchyard G, Phillips A, Grant AD Outcomes following virological failure and predictors of switching to second-line antiretroviral therapy in a South African treatment program. J Acquir Immune Defic Syndr. 2012 11 1;61(3):370–80. 22820803 10.1097/QAI.0b013e318266ee3f PMC3840925

[12] Johnston V, Fielding K, Charalambous S, Mampho M, Churchyard G, Phillips A, et al Second-line antiretroviral therapy in a workplace and community-based treatment programme in South Africa: determinants of virological outcome. PLoS One. 2012;7(5):e36997. 22666338 10.1371/journal.pone.0036997 PMC3362581

[13] Murphy RA, Sunpath H, Lu Z, Chelin N, Losina E, Gordon M, et al Outcomes after virologic failure of first-line ART in South Africa. AIDS. 2010 4 24;24(7):1007–12. 20397305 10.1097/QAD.0b013e3283333639 PMC2902159

[14] Schoffelen AF, Wensing AMJ, Tempelman HA, Geelen SPM, Hoepelman AIM, Barth RE Sustained virological response on second-line antiretroviral therapy following virological failure in HIV-infected patients in rural South Africa. PLoS One. 2013;8(3):e58526. 23505529 10.1371/journal.pone.0058526 PMC3594302

[15] World Health Organization Consolidated guidelines on the use of antiretroviral drugs for treating and preventing HIV infection. 2013 6 Available from: www.who.int 24716260

[16] Labhardt ND, Bader J, Lejone TI, Ringera I, Hobbins MA, Fritz C, et al Should viral load thresholds be lowered? Revisiting the WHO definition for virologic failure in patients on antiretroviral therapy in resource-limited settings. Medicine (Baltimore). 2016 7;95(28):e3985. 27428189 10.1097/MD.0000000000003985 PMC4956783

[17] Ministry of Health of Lesotho, ICF International Lesotho 2014 demographic and health survey. Rockville, MD; 2016 [cited 2016 617] Available from: http://dhsprogram.com/what-we-do/survey/survey-display-462.cfm

[18] Labhardt ND, Keiser O, Sello M, Lejone TI, Pfeiffer K, Davies M-A, et al Outcomes of antiretroviral treatment programmes in rural Lesotho: health centres and hospitals compared. J Int AIDS Soc. 2013;16(1):18616. 24267671 10.7448/IAS.16.1.18616 PMC3838571

[19] Ministry of Health of Lesotho National guidelines on the use of antiretroviral therapy for HIV prevention and treatment. 2013 12.

[20] Labhardt ND, Bader J, Lejone TI, Ringera I, Puga D, Glass TR, et al Is zidovudine first-line therapy virologically comparable to tenofovir in resource-limited settings? Trop Med Int Health. 2015 7;20(7):914–18. 25782332 10.1111/tmi.12509

[21] Howe LD, Galobardes B, Matijasevich A, Gordon D, Johnston D, Onwujekwe O, et al Measuring socio-economic position for epidemiological studies in low- and middle-income countries: a methods of measurement in epidemiology paper. Int J Epidemiol. 2012 6;41(3):871–86. 22438428 10.1093/ije/dys037 PMC3396323

[22] Piot P, Abdool Karim SS, Hecht R, Legido-Quigley H, Buse K, Stover J, et al Defeating aids-advancing global health. Lancet. 2015 6 22;386:171–218. 26117719 10.1016/S0140-6736(15)60658-4

[23] Harries AD, Zachariah R, Lawn SD, Rosen S Strategies to improve patient retention on antiretroviral therapy in sub-Saharan Africa. Trop Med Int Health. 2010 6;15 Suppl 1:70–75. 20586963 10.1111/j.1365-3156.2010.02506.x PMC3059413

[24] Labhardt ND, Sello M, Lejone T, Ehmer J, Mokhantso M, Lynen L, et al Adoption of new HIV treatment guidelines and drug substitutions within first-line as a measure of quality of care in rural Lesotho: health centers and hospitals compared. Trop Med Int Health . 2012 7 29;17(10):1245–54. 22845835 10.1111/j.1365-3156.2012.03051.x

[25] Cohen R, Lynch S, Bygrave H, Eggers E, Vlahakis N, Hilderbrand K, et al Antiretroviral treatment outcomes from a nurse-driven, community-supported HIV/AIDS treatment programme in rural Lesotho: observational cohort assessment at two years. J Int AIDS Soc. 2009 10 8;12(1):23. 19814814 10.1186/1758-2652-12-23 PMC2768674

[26] Rohr JK, Ive P, Horsburgh CR, Berhanu R, Shearer K, Maskew M, et al Marginal structural models to assess delays in second-line HIV treatment initiation in South Africa. PloS One. 2016;11(8):e0161469. 27548695 10.1371/journal.pone.0161469 PMC4993510

[27] Levison JH, Orrell C, Gallien S, Kuritzkes DR, Fu N, Losina E, et al Virologic failure of protease inhibitor-based second-line antiretroviral therapy without resistance in a large HIV treatment program in South Africa. PLoS One. 2012;7(3):e32144. 22427821 10.1371/journal.pone.0032144 PMC3302781

[28] Ciaffi L, Koulla-Shiro S, Sawadogo A, Le Moing V, Eymard-Duvernay S, Izard S, et al., 2LADY Study Group Efficacy and safety of three second-line antiretroviral regimens in HIV-infected patients in Africa. AIDS (London, England). 2015;29(12):1473–81. 10.1097/QAD.0000000000000709 PMC4502989 26244387

[29] Boettiger DC, Nguyen VK, Durier N, Bui HV, Heng Sim BL, Azwa I, et al Efficacy of second-line antiretroviral therapy among people living with HIV/AIDS in Asia: results from the TREAT Asia HIV observational database. J Acquir Immune Defic Syndr. 2015;68(2):186–95. 25590271 10.1097/QAI.0000000000000411 PMC4296907

[30] Chakravarty J, Sundar S, Chourasia A, Singh PN, Kurle S, Tripathy SP, et al Outcome of patients on second line antiretroviral therapy under programmatic condition in India. BMC Infect Dis. 2015;15:517. 26572102 10.1186/s12879-015-1270-8 PMC4647630

[31] Sigaloff KCE, Hamers RL, Wallis CL, Kityo C, Siwale M, Ive P, et al Second-line antiretroviral treatment successfully resuppresses drug-resistant HIV-1 after first-line failure: prospective cohort in sub-Saharan Africa. J Infect Dis. 2012 6;205(11):1739–44. 22448003 10.1093/infdis/jis261

[32] Johnston V, Cohen K, Wiesner L, Morris L, Ledwaba J, Fielding KL, et al Viral suppression following switch to second-line antiretroviral therapy: associations with nucleoside reverse transcriptase inhibitor resistance and subtherapeutic drug concentrations prior to switch. J Infect Dis. 2014 3 1;209(5):711–20. 23943851 10.1093/infdis/jit411 PMC3923537

[33] Boyd MA, Moore CL, Molina J-M, Wood R, Madero JS, Baseline WM HIV-1 resistance, virological outcomes, and emergent resistance in the SECOND-LINE trial: an exploratory analysis. Lancet HIV. 2015;2:e42–e51. 26424460 10.1016/S2352-3018(14)00061-7

[34] Paton N, Kityo C, Thompson J, Bagenda L, Hakim J, Van Ossterhout J, et al Impact of NRTI cross-resistance on second-line PI + NRTI therapy outcomes in Africa. Abstract CROI Conference; 2015 2 23–26 [cited 2015 Sep 4] Available from: http://www.croiconference.org/sessions/impact-nrti-cross-resistance-second-line-pi-nrti-therapy-outcomes-africa (Abstract Number 119).

[35] Rawizza HE, Chaplin B, Meloni ST, Darin KM, Olaitan O, Scarsi KK, et al Accumulation of protease mutations among patients failing second-line antiretroviral therapy and response to salvage therapy in Nigeria. PLoS One. 2013;8(9):e73582. 24069209 10.1371/journal.pone.0073582 PMC3775797

